# Moving toward the automation of the systematic review process: a summary of discussions at the second meeting of International Collaboration for the Automation of Systematic Reviews (ICASR)

**DOI:** 10.1186/s13643-017-0667-4

**Published:** 2018-01-09

**Authors:** Annette M. O’Connor, Guy Tsafnat, Stephen B. Gilbert, Kristina A. Thayer, Mary S. Wolfe

**Affiliations:** 1 College of Veterinary Medicine, Iowa State University, 1800 Christensen Drive, Ames, IA 50011-1134 USA; 2 Australian Institute of Health Innovation, Macquarie University, Sydney, Australia; 3 College of Engineering, Iowa State University, Ames, IA 50011 USA; 4 U.S. Environmental Protection Agency, Research Triangle Park, Durham, NC 27711 USA; 5 National Institute of Environmental Health Sciences, Research Triangle Park, Durham, NC 27709 USA

**Keywords:** Systematic review, Evidence synthesis, Automation, Tools, Priority ranking, Data extraction, Data abstraction

## Abstract

The second meeting of the International Collaboration for Automation of Systematic Reviews (ICASR) was held 3–4 October 2016 in Philadelphia, Pennsylvania, USA. ICASR is an interdisciplinary group whose aim is to maximize the use of technology for conducting rapid, accurate, and efficient systematic reviews of scientific evidence. Having automated tools for systematic review should enable more transparent and timely review, maximizing the potential for identifying and translating research findings to practical application. The meeting brought together multiple stakeholder groups including users of summarized research, methodologists who explore production processes and systematic review quality, and technologists such as software developers, statisticians, and vendors. This diversity of participants was intended to ensure effective communication with numerous stakeholders about progress toward automation of systematic reviews and stimulate discussion about potential solutions to identified challenges. The meeting highlighted challenges, both simple and complex, and raised awareness among participants about ongoing efforts by various stakeholders. An outcome of this forum was to identify several short-term projects that participants felt would advance the automation of tasks in the systematic review workflow including (1) fostering better understanding about available tools, (2) developing validated datasets for testing new tools, (3) determining a standard method to facilitate interoperability of tools such as through an application programming interface or API, and (4) establishing criteria to evaluate the quality of tools’ output. ICASR 2016 provided a beneficial forum to foster focused discussion about tool development and resources and reconfirm ICASR members’ commitment toward systematic reviews’ automation.

## Background

The International Collaboration for Automation of Systematic Reviews (ICASR) is an interdisciplinary group with a shared interest in maximizing the use of technology to aid the transfer of scientific research findings to practice and decision-making. ICASR focuses on automation, rather than specific applications in any particular scientific domain. The group’s aim is to develop the capability for conducting rapid, accurate, and efficient systematic reviews of scientific evidence. Without an automated method for reviewing thousands of research articles, including the many published every year, the findings might be overlooked when developing new policy. Having automated tools for systematic review should enable more transparent and timely review, maximizing the potential for identifying and translating research findings to practical application. As a consequence of this shared goal, the first meeting of ICSAR was held in September 2015 in conjunction with the 23rd Cochrane Colloquium in Vienna, Austria. At that meeting, the group established a set of guiding principles for advancing methods in automation of systematic reviews, referred to as the Vienna Principles (Table 1, http://ebrnetwork.org/the-vienna-principles/). The second meeting was held 3–4 October 2016 in Philadelphia, Pennsylvania, USA.

**Table 1:** Guiding principles proposed at 1st ICASR meeting in Vienna (http://ebrnetwork.org/the-vienna-principles/)

• Systematic reviews involve multiple tasks, each with different issues, but all must be improved.
• Automation may assist with all tasks, from scoping reviews to identifying research gaps as well protocol development to writing and dissemination of the review.
• The processes for each task can and should be continuously improved to be more efficient and more accurate.
• Automation can and should facilitate the production of systematic reviews that adhere to high standards for the reporting, conduct, and updating of rigorous reviews.
• Developments should also provide for flexibility in combination uses, e.g., subdividing or merging steps and allowances for different users to use different interfaces.
• Different groups with different expertise are working on different parts of the problem; to improve reviews as a whole will require collaboration between these groups.
• Every automation technique should be shared, preferably by making code, evaluation data, and corpora available for free
• All automation techniques and tools should be evaluated using a recommended and replicable method with results and data reported.

The goals of the second ICSAR meeting were to (1) facilitate discussion within the community and foster collaboration among different stakeholders, (2) gather viewpoints on the progress toward automation made to date and outstanding challenges, and (3) gather opinions on approaches to potential solutions that might guide future projects. The meeting sought to include the spectrum of domains necessary to achieve these goals, including users of summarized research, methodologists, and technologists. Users include producers and consumers of systematic reviews from any domain that uses research reviews. Methodologists are those who explore the production processes and quality of systematic reviews. Technologists are software developers, statisticians, and vendors with skill sets not necessarily specific to automation of systematic reviews, although most have applied their skills and expertise to this problem domain in the past. That some participants naturally fit into one group was acknowledged, while others straddle two or all three.

### The second ICASR meeting: scope

The second ICASR meeting began with a discussion of the scope of ICASR. This discussion affirmed the premise that ICASR seeks to apply research synthesis methods to all areas of science. Scientific areas of interest to participants included clinical health, public health, preclinical research, food production, ecology, wildlife, and environmental health. Many disciplines in the scientific community that synthesize research and use the systematic review methodology are not currently represented in ICASR. ICASR should seek to engage those disciplines, and those community members should be given the opportunity to adopt or adapt automation tools as needed.

The role of ICASR in tool development was discussed. As used here, “tool” refers to a software application with a user interface that fully or partially automates a task conducted by systematic reviewers. It is thus distinct from an algorithm (which might be embedded in a tool), in that the tool is accessible and usable by people without programming skills. The technologists indicated that development of scalable and generalizable tools is a standard for the industry, with the aim of having common tools that can be tailored as needed. The tailoring of tools is a challenge for the community, because many developers and funders focus on tools for specific tasks. Converting, adapting, and validating those tools to work on the same task in other areas can seem duplicative. The goal of the collaboration, however, is not to develop or support a particular synthesis pipeline or set of tools but rather to create a community where multiple tools can be shared with the goal of more rapid progress.

Meeting participants were presented with the idea that ICASR should foster the development of a system that would connect individual automation tools in a system of shared protocols. This system and integration would promote open collaboration between groups (some working on interoperability “backbone” systems, some on individual tools). Interconnectedness was considered critical for rapid progress. For tool developers, the advantages are rapid adoption and adaptation of domain-specific tools to other scientific domains. Such an approach maximizes the reach of tools. For domain-specific users, the advantage of interconnectedness is the ability to adopt and adapt tools developed by others.

Further to the guiding principles the Vienna Principles embody, the participants proposed that the technical principles for the ICASR would also include:

- The collaboration considers that reviews realistically would include manual, semi-automated, and fully automated tasks in a single workflow.
- The collaboration welcomes development of multiple tools that perform the same task in different ways with the goal that end-users would be able to choose the most appropriate tool to use for each review.
- The collaboration would, through experience, agree on an open-source API (application programming interface), such that development of new tools would contribute to shared, interconnected systems.
- The collaboration welcomes proprietary and open-source tools and systems. The goal, however, is to have APIs that are consistent so users can opt in and out of using any tool that can exchange data seamlessly with other tools.
- The collaboration seeks to focus on approaches to integrate tools, rather than on particular tools for particular steps or a particular pipeline for a discipline.

### The second ICASR meeting: purpose

The goal of the meeting was to:

- Identify common needs of users in all domains.
- Identify common challenges to meeting those needs.
- Identify potential approaches to common challenges.
- Identify what is needed to make near-term, rapid progress.
- Deliver products rather than just raise awareness.

The first goal of the meeting was to identify common needs for automation across scientific domains and the common challenges in meeting those needs. The approach to addressing this issue was threefold. First, paired participants discussed their needs and challenges. Then, groups of five to seven participants were asked to consider all presented information and to identify, consistent with the goal above, issues that would enable rapid and broad progress if they could be resolved. The third task occurred on the second day of the program, which aimed to identify how to meet the challenges to near-term progress. Three groups were formed, and project leaders guided group discussion on specific topics with identification of short-term goals (6 months) and longer-term goals (about 3 years). The project topics related to prototyping a system for maintaining “living” systematic reviews, increasing efficiency of citation screening via a pipeline of multiple tools, and integration across platforms via an extraction dashboard. These discussions were then shared across all groups for discussion and critique.

### Common needs and challenges for systematic reviews across scientific domains

Several challenges were identified during the meeting (Table 2). Some challenges are somewhat philosophical and relate to technology acceptance. More immediate challenges are (1) communication issues between groups that are developing tools, (2) accurate data extraction, and (3) translation of available technology to tools with user interfaces that would allow users to incorporate them into realistic workflows. Although participants recognized that many tasks in the systematic review process need tools, an interesting gap between technologists and users was noted. Technologists did not consider semi-automating the identification of relevant studies a current challenge, as this is currently in practice in software packages such as Abstrackr and Eppi Reviewer Software.^1^ However, the state of the art is not such that producers of systematic reviewers routinely use these systems, and several explanations were voiced. Two are a lack of transparency by machine-learning systems and a shortage of studies showing the benefits of screening systems in a variety of scientific disciplines. Of the remaining tasks or steps, accurate data extraction of study characteristics was identified as a current challenge by algorithm developers. Other tasks, such as automated reporting using general tools for reproducible research and reporting (e.g., Rmarkdown and knitR^2^) or specialized packages (e.g., RevMan HAL^3^), were considered important, although less technically challenging.^4^

**Table 2:** Challenges to automation identified by meeting participants and invited speakers

Broader challenges
• Social acceptance of automation technology
• Development of flexible systems for different disciplines
• Acquiring resources for development
• Fostering collaboration in a competitive environment
• Keeping up with rapidly evolving technologies and approaches, such as open data
• Making automation approaches compatible with stakeholder transparency needs, that is, the “black box” nature of many technologies such as machine learning
Technological challenges
• Designing an application programming interface that meets the needs of multiple scientific domains and goals for different systematic reviews
• Integrating an application programming interface into both new and existing software tools
• Creating cross-compatibility of tools
• Addressing issues of intellectual property
• Meeting review-specific/data-specific challenges
• Extracting data from full texts
• Developing approaches for algorithm and tool validation

### Toward solutions: possible approaches

The workshop identified several short-term projects to advance the goal of automating tasks in the systematic review workflow.

### Comprehension of required tasks/steps

A more comprehensive understanding is needed of the processes required to complete the tasks or steps in a systematic review. Also needed is knowing what tools could be made available for users to complete those tasks, that is, a story-board for conducting a review. Such information would facilitate a common approach to tool development and could improve communication with funders, producers of systematic reviews, and developers. As the producers of systematic reviews seeks to create user-friendly tools, developers should have comprehensive, standard use-cases, to ensure users can seamlessly adopt the tools. For example, a use-case for relevance screening would list the actions or events that define the interactions between the producers of systematic reviews and any software systems (such as search engines, reference managers, and screening tools) to achieve a goal of identifying relevant studies. From the time the protocol for the systematic review is conceived to the time the review is updated, having use-cases documenting the needs of all users, where available, for the developers working in the ICASR would be most efficient.

### Validation of tools

Promoting the validation of tools is essential. One approach is to develop and maintain validated datasets that tool developers could use; however, such datasets are often limited in scope, because they are validated only for the original investigator’s purpose. Strategic compilation of “gold-standard” annotated datasets covering a range of topics would be ideal to ensure sufficient variation in training and validation datasets. An essential component of validation is to define what constitutes a minimal validation standard.

### Development of data extraction tools

A widely recognized need is to develop data extraction tools that would enable annotation of full texts, extraction of data and information to assess risk of bias, and transfer of data to next steps in review tasks. This area, in particular, would benefit from further collaboration between users and developers. Currently, no agreement has been reached on which elements to extract, how accuracy would be measured, and how these data can be shared in the collaboration. Further hindering this issue are questions around copyright and permission to share annotated documents.

### Interoperability standard

Participants agreed that having a standard method for tools to interoperate would be beneficial. In particular, standardization would allow users to readily understand the tools, reducing the effort needed for developing algorithms and incorporating them into tools. The main method for tool interoperability discussed was an API that can manage references and the data they contain so that these can be passed from one tool to another.

Systems that host the API and provide it to other tools are called “backbone” systems. Individual tools that implement the API could then be used as part of any backbone system.

### Quality criteria

Criteria to assess the quality of the output using automated tools are needed for systematic reviews to be generally accepted. This point touches on a broader need: methods to evaluate the quality of systematic reviews in general. ICASR will endeavor to work with the broader community to develop quality indicators for systematic reviews.

Additionally, workshop participants recognized that acceptance of automated systematic review will require cultural changes. Societal issues, considered outside of ICASR’s scope, were not discussed. ICASR members believe that fostering discussion and collaboration among stakeholders who create and use systematic reviews, and among publishers and journal editors, will be vital to the advancement and acceptance of automation.

## Conclusion

ICASR 2016 provided a beneficial forum for engaging a range of disciplines and expertise in focused discussion about tool development and resources to advance the automation of systematic reviews. The meeting highlighted challenges, both simple and complex, and raised awareness among participants about ongoing efforts by various stakeholders. ICASR hopes to foster continued interaction and communication among stakeholders as efforts in systematic review automation progress.

## Footnotes

1 http://eppi.ioe.ac.uk/cms/Default.aspx?alias=eppi.ioe.ac.uk/cms/er4.

2 Xie, Yihui. Dynamic Documents with R and knitr, Second Edition. 2013. Chapman & Hall/CRC The R Series. ISBN-13: 978-1498716,963. ISBN-10: 1498716,962.

3 http://schizophrenia.cochrane.org/revman-hal-v4.

4 Influences of Reproducible Reporting on Work Flow. http://chance.amstat.org/2015/11/reproducible-reporting/.

